# Integrating parental genomes to reduce reference bias and identify intramuscular fat genes in Qinchuan Black pigs

**DOI:** 10.1186/s40104-025-01236-3

**Published:** 2025-07-20

**Authors:** Guangquan Lv, Peiyu Yang, Ao Guo, Minghao Cao, Dong Li, Zhe Liu, Mingyu Wang, Jingchun Sun, Rongrong Ding, Taiyong Yu

**Affiliations:** 1 Key Laboratory of Animal Genetics, Breeding and Reproduction of Shaanxi Province, Laboratory of Animal Fat Deposition & Muscle Development, College of Animal Science and Technology, Northwest A&F University, Yangling, Shaanxi 712100 China; 2 Kunming Institute of Zoology, Chinese Academy of Sciences, Kunming, Yunnan 650201 China; 3 Key Laboratory of Agroecological Processes in Subtropical Region, Institute of Subtropical Agriculture, Chinese Academy of Sciences, Changsha, 410125 China

**Keywords:** Genotyping, Intramuscular fat, Parental genome, Pig

## Abstract

**Background:**

Traditional genomic analysis relies on a single reference genome, which struggles to effectively characterize the genetic diversity among populations. This is due to the substantial genetic differences between the genome of the studied species and the reference genome, potentially introducing reference bias.

**Results:**

In this study, we focused on Guanzhong Black pigs (GZB), Danish Large White pigs (DLW), and their hybrid offspring, Qinchuan Black pigs (QCB). We provided two high-quality parental genomes at the chromosomal level and constructed a parental genomic reference panel to detect SNPs (single nucleotide polymorphisms), INDELs (insertions and deletions), and SVs (structural variations). Compared with the single-reference method, the integrated parental genomic strategy identified 5.48% more SNPs and 67.84% more INDELs. The uniformity of variant distribution and genome functional annotation remained consistent before and after integration, while the ratio of non-reference/non-reference genotypes was also improved. In population genetic structure analysis, principal component analysis (PCA) of the three variant types (SNPs, INDELs, and SVs) exhibited good clustering effects, and ADMIXTURE analysis demonstrated consistent stratification. Selection signal analysis based on the integrated parental genomic strategy successfully identified more differentiated windows and positively selected genes. By leveraging multiple variant types and employing two selection signal methods, we jointly identified several novel intramuscular fat candidate genes (*MSMO1*, *SMC6*, *CCDC158*, *KIT*, *CCNC*, etc.), which could not be identified by the single-reference method alone. Functional validation of the gene *MSMO1* revealed its role in promoting intramuscular adipocyte proliferation and inhibiting adipogenic differentiation.

**Conclusions:**

This study is the first to construct a parental genomic reference panel specifically for pig hybrid populations, which significantly reduces reference bias and exhibits superior performance in downstream analyses. This strategy offers new possibilities for genomic selection breeding of livestock and establishes a methodological foundation for precisely dissecting complex traits in hybrid populations.

**Supplementary Information:**

The online version contains supplementary material available at 10.1186/s40104-025-01236-3.

## Background

Long-read sequencing has revolutionized genomics, offering critical insights into genome complexity, structural variations, and genetic diversity [1]. Unlike short-read sequencing, which may have difficulty processing repetitive regions and miss some hidden variants (long fragment insertions, methylation modifications, etc.) [2–4]. Long-read sequencing is conducive to improving the continuity, accuracy, and scope of variant phasing [5], thereby achieving more accurate de novo assembly and mapping to more genetic variations. This progress is particularly valuable in the field of animal genetics, where high-quality genome assembly can better identify previously undiscovered variants associated with desired traits [6–10]. In recent years, with the advancement of bioinformatics tools, a variety of new methods have been proposed that have significantly improved the performance of long-read sequencing in variant detection and data processing, making it an extremely powerful tool for exploring genomes [11].

A key challenge in population genomics is the reliance on a single reference genome, which can introduce biases when studying populations that diverge greatly from the reference genome, particularly in highly diverse individuals [12, 13]. For populations with large phylogenetic distances or rich genetic diversity (e.g., hybrid populations), analyses based on a single reference can obscure variant frequencies and mask important genetic information [14, 15]. Because single-reference approaches often underestimate or simplify genetic diversity, they fail to fully capture the unique variation in a population’s genome. Utilizing a population-specific reference genome can reduce these biases, improve the reliability of genetic variant calls, and enhance downstream analyses [16]. Ideally, for hybrid populations, genotyping that incorporates both parental genomes would capture all genetic variation segregating within the population [17], creating a comprehensive reference framework for more efficient dissection of the genetic basis of complex traits.

Qinchuan Black pig (QCB) is a hybrid breed derived from the crossing of Guanzhong Black pig (GZB) and Danish Large White pig (DLW). Renowned for its high-quality meat and intramuscular fat content [18], QCB holds significant research value. Given the notable genetic differences between its parental breeds, traditional single-reference genome-based analyses may encounter substantial limitations. The strategy of integrating both parental genomes holds great promise in the genomic studies of QCB. By combining the genomic information of the two parents, it may potentially enhance the comprehensiveness of genetic variation detection. Since the genetic composition of QCB is a combination of the genomes of two distinct breeds, using a traditional single-reference strategy may overlook many breed-specific and hybrid-related genetic variations. The strategy of integrating both parental genomes may be able to identify these concealed variations, which is crucial for dissecting the genetic mechanisms underlying complex traits in QCB. Furthermore, by incorporating the genomic information of both parents, this strategy may effectively improve the completeness of variation detection and the accuracy of genotyping. It also aids in revealing the genomic characteristics of various types of variations, such as Structural Variation (SVs) and Small Insertion and Deletion (INDELs).

In addition to the research value of the QCB breed itself, the integration strategy of both parental genomes also has broad application potential in the entire livestock genomics. For genome selection and genetic map construction in livestock breeding, this method can improve the resolution of complex traits, especially in cross-breeding projects, and is expected to significantly reduce bias in genotyping, thereby improving breeding accuracy. By integrating the genomes of both parents, breeders have the potential to more accurately capture genetic variations associated with key traits such as disease resistance, rapid growth, and high meat quality, providing a scientific basis for selecting desirable traits in the breeding process and thereby effectively supporting future selective breeding efforts.

## Materials and methods

### Sample information

Regarding the breeding of QCB pigs, in the early stage, through phenotypic comparison and other analyses, GZB sows (*n* = 200) and DLW boars (*n* = 21) were selected as the F0 generation parental stock. By crossing GZB♀ (sows) × DLW♂ (boars), an F1 generation heterozygous population was obtained. From the F2 generation to the F4 generation, pigs from different families within each generation were mated, and superior individuals were selected for the next generation based on growth, reproductive and other relevant indicators. Starting from the F5 generation, based on the collected phenotypic data, genomic selection (GS) and other analytical methods were employed to conduct closed-group breeding among superior individuals, gradually fixing favorable haplotypes. Up to now, the QCB pigs have been bred to the F6 generation.

Fresh blood was collected from a GZB sow at the Guanzhong Black Pig Core Breeding Farm (Xianyang, Shaanxi Province, China) as the DNA source for generating de novo genome assembly sequence data. In addition, we collected genomic data from 40 DLW pigs, 31 GZB pigs, and 37 QCB pigs. The DLW pigs and GZB pigs all originated from the same F0 generation population or their offspring, and the QCB pigs were all from the F5 generation. These re-sequencing data were downloaded from the NCBI Sequence Read Archive (SRA; https://www.ncbi.nlm.nih.gov/sra/; Table S1).

### Genome sequencing

For cycle consensus sequencing (CCS), SMRT bell libraries (15 kb) were constructed according to the PacBio protocol and sequenced on the PacBio Sequel II (e) platform to generate HiFi reads. A total of 10,168,284 reads were generated with a coverage of 35.42 ×. For short-read DNA sequencing, the sequencing library was constructed according to the MGISEQ protocol and sequenced on the DNBSEQ-T7 platform, with paired-end sequencing of 150 bp.

### Genome assembly and annotation

For the genome of GZB pig, we used a fast haplotype-resolved de novo assembler, Hifiasm (v0.16.0; with default parameters) [19] to assemble the PacBio HiFi reads. To generate non-redundant genome sequences, the haplotigs and contig overlaps in the initial assemblies were removed using the purge_dups program, which is based on PacBio read depth and a K-mer counting strategy. The primary contig assembly was scaffolded to chromosome level using RagTag (v2.0.1) [20] with the reference genome assembly (Sscrofa 11.1). Repetitive sequences were identified de novo from the genome of GZB pig using RepeatModeler (v2.0.1) [21]. Then, RepeatMasker (v4.1.1) [22] was used to mask the genome using the de novo repeat library and Repbase (v22.11; https://www.girinst.org/repbase/) [23]. Genome annotation was performed on the repeat-masked pseudochromosome genome assembly using Liftoff (v1.6.1) [24] based on the reference genome. Functional annotation was achieved by comparing predicted proteins against public databases with an E-value threshold of 1e-5, including NCBI non-redundant protein sequences database (Nr), SwissProt, and TrEMBL using Diamond BLASTP (v2.0.11.149) [25]. QUAST (v5.0.2) [26] and BUSCO (v5.2.2) [27] were used with the vertebrate odb10 database to assess genome assembly quality and completeness.

### Single reference genotyping

We genotyped 108 high-depth (average sequencing depth 16.6 ×) samples (Table S1). Adapters and low-quality reads were filtered out using fastp (v0.20.0) [28] with default parameters. Clean reads were mapped to the reference genome (Sscrofa 11.1) using BWA-MEM (v0.7.17) [29] with default parameters. SNPs (single nucleotide polymorphisms) and INDELs (insertions and deletions) calling was performed using the GATK pipeline [30] and HaplotypeCaller models.

### Construction of pan-genome graphs and graph-based genotyping

The DLW genome has been assembled in previous research studies [10]. The reference genome, the GZB pig genome, and the DLW pig genome were visualized using NGenomeSyn (v2.0) [31] and Minimap2 (v2.28) [32], demonstrating high collinearity of most chromosomal regions.

We ran the minigraph-cactus pipeline using the cactus (v2.4.2) [33] Docker image and the nextflow pipeline (https://github.com/WarrenLab/minigraph-cactus-nf) built for this purpose. We took the GZB pig genome and the DLW pig genome as input. We specified Sscrofa 11.1 as the reference because, while it is not the highest quality assembly, it is the best RefSeq annotated assembly.

The base-level pan-genome map generated above was preprocessed using the genotyping pipeline (https://bitbucket.org/jana_ebler/hprc-experiments/src/chm13-based-pipeline/genotyping-experiments/). The three genomes were subsequently integrated into the biallelic VCF generated here. We genotyped a total of 108 samples from the hybrid population using PanGenie (v3.00) (Table S1) [34]. The integration of variant sets was based on single reference genotyping and graph-based genotyping. Prior to this, we removed a small number of multi-allelic variants and variants containing deletions. For overlapping sites, variants with a genotype consistency exceeding 90% were retained. The following variants were excluded: those with a proportion of missing genotypes greater than 10%, and those with a minor allele frequency (MAF) lower than 5%. For both SNPs and INDELs, variants that deviated from Hardy–Weinberg equilibrium (HWE) with a significance threshold set at *P* < 0.000001 were also removed.

The variants were further annotated and classified into SNPs, INDELs, other synonymous or non-synonymous variants, intronic variants, and variants located in upstream or downstream regions of genes or intergenic regions using VEP (v110.0) [35].

### Phylogenetic and population genomic analyses

We utilized variants that had undergone quality control procedures and employed VCFtools (v0.1.16) [36] and Plink (v1.90) [37] software to convert the VCF-formatted file into Plink binary format. Subsequently, we used the top two principal components (PCs) to assign the 108 pig accessions to PCA clusters. To elucidate the genetic ancestry and the admixture patterns among breeds, further population structure was investigated using ADMIXTURE (v1.3.0) [38] with the optimal population size chosen from K = 2 as that with least error after resampling for cross-validation.

### Selective scan analysis

Here, variants were filtered based on the criteria of a missing genotype proportion less than 10% and a minor allele frequency (MAF) below 0.05. Fst values and XP-CLR scores between QCB pigs and DLW pigs were calculated using VCFtools and the XP-CLR package (v1.1.2) [39] with a sliding window of 50 kb and a step size of 25 kb. Results were visualized using the qqman R package (v0.1.4) [40]. The top 5% of regions were designated as candidate selection regions, and the genes in these regions were considered candidate genes. Candidate selected regions were mapped to Animal QTLdb for QTL annotation [41].

We conducted a genome-wide association study (GWAS) based on an integration strategy to identify candidate loci under selection. The approach employed was EigenGWAS [42], which utilized the eigenvector (the first principal component; PC1) derived from principal component analysis (PCA) as the "phenotype" to analyze quality-controlled SNPs, INDELs, and SVs from 37 QCB pigs and 40 DLW pigs. The fact that PC1 clearly distinguished QCB pigs from DLW pigs supported the application of EigenGWAS (Fig. S4). SNPs, INDELs, and SVs with Bonferroni-corrected *P*-values less than 0.05/n were considered significant loci. We performed gene annotation for the regions 100 kb upstream and downstream of the loci.

### Isolation of porcine intramuscular preadipocytes

All experiments were conducted in accordance with relevant guidelines and regulations. Select a healthy 3-day-old male Qinchuan Black piglet (F5 generation), washed in warm water and surface sterilized with 75% ethanol. The longest dorsal muscle was taken and placed in a petri dish under aseptic conditions and rinsed twice with PBS containing 2% penicillin–streptomycin (2% penicillin–streptomycin: PBS = 2:100). The fascia of the longest dorsal muscle was removed and then cut into small pieces and placed into a 50-mL centrifuge tube to which trypsin was added and the tissue was digested on a water bath shaker at 37 °C until the tissue was loosened. At the end of digestion, the tissue was allowed to settle and the supernatant was collected in a centrifuge tube after being filtered through 70 mesh and 200 mesh filters and centrifuged at 1,800 r/min for 10 min and the supernatant was discarded. The cells were washed by adding PBS to the centrifuge tube, centrifuged at 1,500 r/min for 5 min, the supernatant discarded, the cells were resuspended by adding complete medium, and inoculated into Petri dishes and incubated at 37 °C for 2.5 h at 5% CO_2_, the supernatant discarded and replaced with complete medium for further incubation.

### Cell culture and differentiation of pig intramuscular adipocytes

We used DMEM:F12 = 1:1 medium (DMEM Hyclone, Logan, UT, USA) to culture pig intramuscular adipocytes. The medium was supplemented with 10% fetal bovine serum and 2% penicillin–streptomycin. Cells were cultured in an incubator environment at 37 °C with 5% carbon dioxide until the cells grew to the point of contact inhibition. After 2 d of contact inhibition, the cells were switched to differentiation induction medium I (10 µmol/L insulin, 1 µmol/L DEXA, 0.5 mmol/L IBMX, 1 µmol/L rosiglitazone) (Sigma, Shanghai, China) for 2 d. The culture was then switched to induction medium II (10 µmol/L insulin) for 6 d and the medium was changed every 2 d until convergence of fat droplets was seen.

### RT-qPCR and Western blot

Total RNA was extracted from pig intramuscular adipocytes using TRIzol reagent (Takara Bio, Otsu, Japan) according to the manufacturer’s instructions. cDNA was reverse transcribed from RNA using PrimeScript RT kit (Takara Bio, Otsu, Japan). Real-time quantitative polymerase chain reaction (RT-qPCR) was performed using a SYBR premixed Ex Taq kit (Vazyme Biotech, Nanjing, China). All primers are shown in Table S2.

After rinsing the cells with PBS, total protein was extracted from the supernatant using RIPA Lysis Buffer (Beyotime, Shanghai, China) containing phosphatase inhibitor A, phosphatase inhibitor B, and protease inhibitors. Protein samples were separated, and then transferred to a polyvinylidene difluoride (PVDF) membrane (Millipore, Bedford, MA, USA). After rinsing 3 times with 1 × TBST, secondary antibody (IgG 1:1,000, Goat Anti-Rabbit IgG, Boster, BA1039; Boster Biological Technology, Pleasanton, CA, USA) was added and incubated for 1.5 h at room temperature. Immunoblots were scanned using a gel imager (Bio-Rad, USA) and the lightness and darkness of the bands were analyzed using an image analysis application (ImageJ).

### Statistical analysis of data

GraphPad (v8.3.0) software was used for significance analysis and multiple comparison. For the comparison between two groups, an independent-samples t-test was conducted. *P* < 0.05 was defined as the significance threshold. RT-qPCR data through the 2^-ΔΔCt^ method analysis.

## Results

### GZB pig genome assembly, genome characterization, and genome variation

For de novo assembly of the GZB pig genome, we used PacBio long-read sequencing (35.4 × coverage) to generate a chromosome-scale genome assembly for GZB pig (Table S3 and S4). The contig N50 length of the GZB pig genome is 42.07 Mb, which was similar to the official reference genome assembly (Sscrofa 11.1; 48.2 Mb) (Table 1) [43]. Evaluation of base accuracy and completeness of the genome assembly using Quast [26] and BUSCO [27] indicated the high quality of the genome assemblies (Table 1).

**Table 1:** Summary statistics of pig genome assemblies

Genome assembly	GZB	Sscrofa11.1
	(This study)	Warr et al. [43]
Species	Guanzhong Black	Duroc
Assembled genome size, bp	2,676,082,270	2,501,912,388
No. contigs	6,926	1,117
No. scaffolds	156	705
TE elements, %	25.27	26.8
Predicted protein coding gene number	20,813	20,661
Mean gene length, kb	51.14	50.94
Contig N50, bp	42,067,722	48,231,277
Scaffold N50, bp	138,839,465	88,231,837
Complete BUSCOs (Genome)	98.0%	97.7%
Complete BUSCOs (Protein)	91.7%	97.6%

The percentage of repetitive sequences was 43.1% in the GZB pig genome (Table S5). Combining evidence from ab initio predictions and protein homology, a total of 20,813 protein-coding genes were predicted in the GZB pig genome. BUSCO analysis showed that the predicted genes in the GZB pig genome captured 91.7% of the core conserved animal genes (Table 1).

**Fig. 1:**
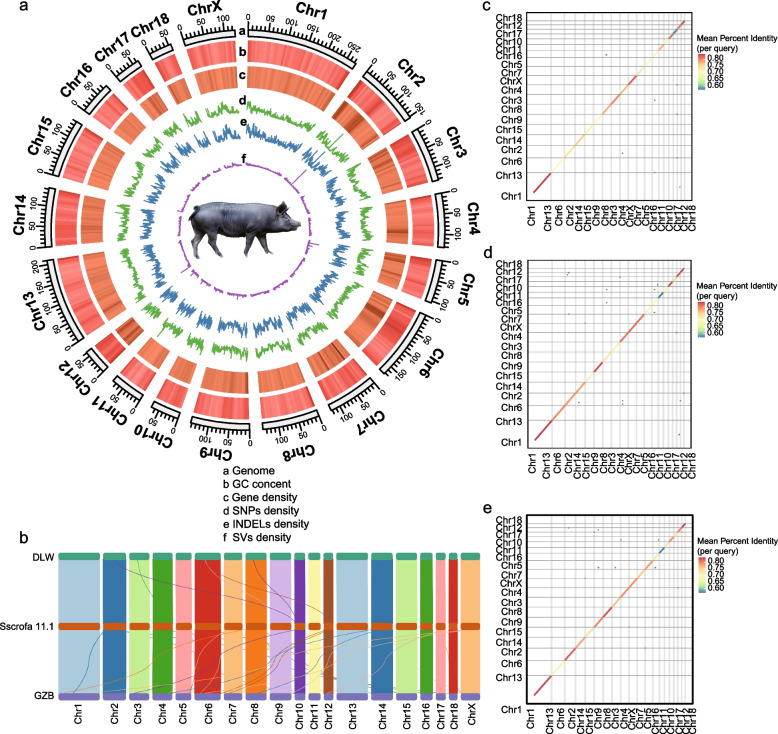
Guanzhong Black pig genome assembly and genome homology and colinearity analysis. **a** Circos displays important features of the assembled Guanzhong Black pig genome and pan-genome. The name and size of the chromosome (a), GC content (per 500 kb window) (b), Protein coding gene density (per 500 kb window) (c), Single nucleotide polymorphisms (SNPs) in the pan-genome (SNPs score per 500 kb window) (d), Small insertions or deletions (INDELs) in the pan-genome (INDELs score per 500 kb window) (e), Large structural variations (SVs) longer than 50 bp in the pan-genome (SVs score per 500 kb window) (f). **b** Synteny plot of DLW pig, Sscrofa11.1 and GZB pig genomes. **c** Genomic homology map of Sscrofa11.1 reference genome and GZB pig genome. **d** Genomic homology map of Sscrofa11.1 reference genome and DLW pig genome. **e** Genomic homology map of the DLW pig genome and the GZB pig genome

The three genome assemblies showed significant differences in the number, type, and relative abundance of transposable elements (TEs) (Table S5). The most common TE class found in the pig genome corresponds to LINE (long interspersed nuclear element) retrotransposons (Table S5). We observed a high level of colinearity between the parental genome of QCB pigs and Sscrofa 11.1, and there may be more insertions, deletions, or rearrangements on some chromosomes (Fig. 1b–e).

### Integration and comparative analysis of genotyping results based on traditional single reference genome and two parental genomes

We used Sscrofa 11.1 as a reference and constructed a small pan-genome containing Sscrofa 11.1 and the parental genomes of QCB pigs based on whole-genome alignment (Fig. S1). We found that there were some large structural variations between the parental genomes of QCB pigs and Sscrofa 11.1 (Table S6). A total of 10,649,404 SNPs, 3,216,185 INDELs and 61,078 SVs were identified from the pan-genome, and the distribution of SNPs and INDELs on the genome was similar (Fig. 1a). Among them, INDELs and SVs were integrated into the reference genome, resulting in a pan-genome containing a total of 28.59 Mb of sequences not present in the reference genome. Of these sequences, 15.50 Mb and 14.67 Mb came from the GZB pig and DLW pig genomes, respectively, and 2.27 Mb came from two germplasms (Table S6).

Initially, we used the traditional single reference genome strategy to genotype 108 high-depth samples from the QCB pig hybrid population (Fig. S1a). We then merged the single reference genome with the graph-based genotyping results (Fig. S1c). We set the identifier for single reference genome typing as SR, the identifier for graph-based genotyping as Pan, and the identifier for the merger of the two as Merged.

The SR method is implemented based on tools such as BWA (Burrows-Wheeler Aligner) and GATK (Genome Analysis Toolkit). This method usually involves aligning short read sequences to a fixed reference genome and calling SNPs and INDELs by identifying variant sites in the alignment. Although this strategy performs well in the detection of SNPs and INDELs, its reliance on a single reference genome may lead to reference bias, especially in regions with high repetitiveness and variation in the genome.

PanGenie, which we used in the Pan method, is an accurate and efficient genotyping algorithm that improves genotyping performance by utilizing the pan-genome reference and k-mer counts of haplotype resolution, and is particularly outstanding in variant identification in large insertion and repeat regions [34]. We input the variant set in the upstream pan-genome into PanGenie, and use it as a graph-based typing panel to type the population reads.

The SR method can call variants from population reads, while the Pan method is only based on the variant set obtained during the construction of the pan-genome. In the case of a closed hybrid population, the variants detected by the SR method will increase with the increase in the number of samples and gradually reach saturation. The results of the Pan method typing directly depend on the variant set obtained during the construction of the pan-genome, and are less correlated with the number of population samples. This difference makes the results full of diversity and uncertainty.

**Fig. 2:**
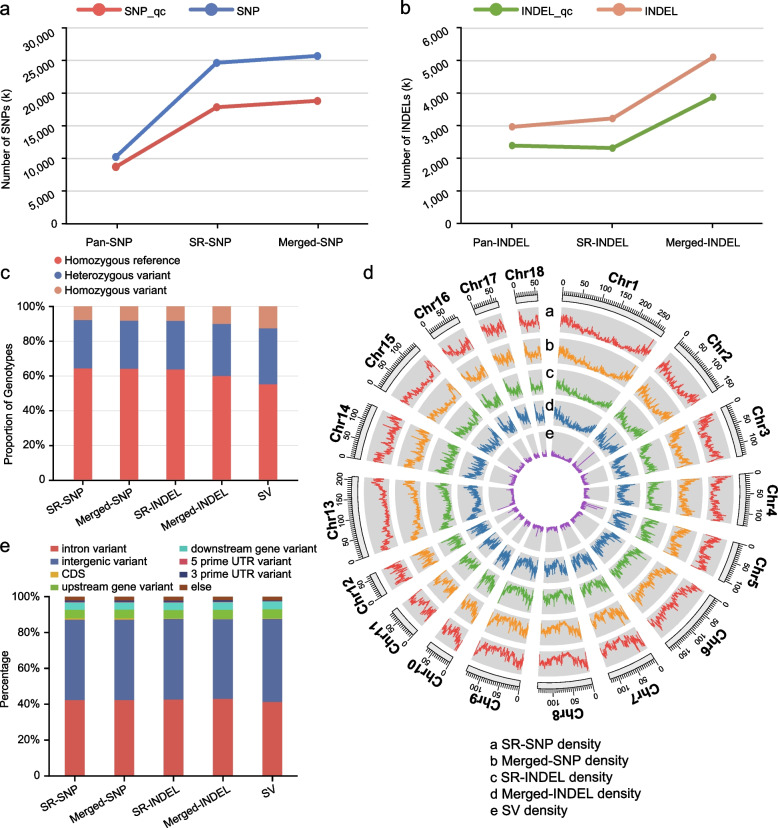
Comparison of variant set features obtained by different strategies. The sources of the three variant sets: (1) Pan, a graph-based method using a pan-genome, (2) SR, a single reference method using Sscrofa 11.1, and (3) Merged, a variant set integrating (1) and (2). **a **and** b** The line charts exhibit comparisons of the number of single nucleotide polymorphisms (SNPs) (**a**) and small insertions/deletions (INDELs) (**b**) before and after quality control using the three strategies. **c** The bar charts summarize the frequencies of different genotype types for SNPs, INDELs, and structural variations (SVs). **d** Circos shows the density distribution of SNPs, INDELs, and SVs on the whole genome. Annotation results of SNPs, INDELs, and SVs.** e** The bar charts display the annotation results for SNPs, INDELs, and SVs

In order to reduce the false positives caused by sequencing data in the SR method and improve the typing effect, we used high-depth (average depth of 16.6 ×) sample data (Table S1). After excluding multi-allelic sites and sites with missing sample genotypes, we counted the number of different variant types before and after quality control (Fig. 2a and b; Table S7), and the number of variants increased after merging (4.26% increase in SNPs and 58.40% increase in INDELs before quality control; 5.48% increase in SNPs and 67.84% increase in INDELs after quality control), which was different from the expected increase ratio for SNPs and INDELs. The increase ratio of the total number of variants after merging was 2.12% higher after filtering (12.65%) than the original (10.53%), which was caused by the small change of the variant set obtained by the pan method before and after quality control.

After merging the variants obtained by the two methods, the proportion of calls for both heterozygous reference/non-reference and non-reference/non-reference genotypes increased (Fig. 2c; Table S8). INDELs had a higher proportion of heterozygous reference/non-reference and non-reference/non-reference genotypes than SNPs, while SVs had the most non-reference/non-reference genotypes (Fig. 2c; Table S8). This suggests that the method based on the two parental genomes not only improves the detection ability of variants, but also that the diversity of SVs and INDELs is richer which may reflect the genetic variability and selection pressure of these variants in the population.

We compared the density of SNPs and INDELs obtained by the SR method, SVs obtained by the Pan method, and SNPs and INDELs obtained by merging the two methods on the whole genome (Fig. 2d). For SNPs and INDELs, the uniformity of the variant density after merging was generally improved, which may be because the typing based on the two parental genomes supplemented the insufficient detection of traditional single reference methods in some areas and reduced the influence of "reference bias". The annotation results showed that regardless of the type of variation, most of them were located in intergenic or intronic regions, and the proportional distributions of different variant types before and after data integration exhibited a high degree of consistency (Fig. 2e; Table S9). This reflects that the introduction of the two parental genomes did not significantly change the functional annotation distribution of variation types, but expanded the variation detection in these regions.

### Population structure

**Fig. 3:**
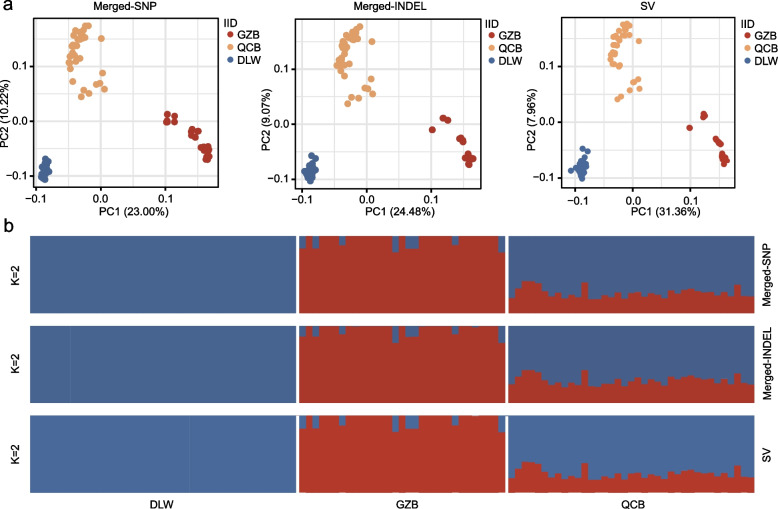
Population genomic analysis was performed on 108 pigs. **a** Principal component analysis (PCA; PC1 and PC2) was performed on 108 samples based on the integrated variant set. **b** ADMIXTURE analysis was performed based on the integrated variant set (K = 2)

To validate the integration strategy, we performed a population structure analysis to verify that the population structure was consistent with the structure inferred from the traditional single reference genome strategy. The principal component plot shows that all individuals from the same breed cluster together, the clustering in the principal component plot based on SNPs became more pronounced after applying the integration strategy. (Fig. 3a; Fig. S2a). Both before and after applying the integration strategy, the percentage of variance explained by INDELs was higher than that by SNPs. Moreover, the percentage of variance explained by PC1 for SVs was greater than that for both SNPs and INDELs, which highlights the potential of INDELs and SVs for population genetic structure analysis. Cluster analysis based on maximum likelihood estimation divided the samples into three independent clusters: GZB pigs, DLW pigs and QCB pigs (Fig. 3b; Fig. S2b). Our results show that the population structures derived from SNPs, INDELs and SVs are similar. QCB pigs each possess more than half DLW pig ancestry, which is likely a result of artificial selection during the breeding process.

### Integrative strategy reveals novel candidate genes for intramuscular fat in pigs

**Fig. 4:**
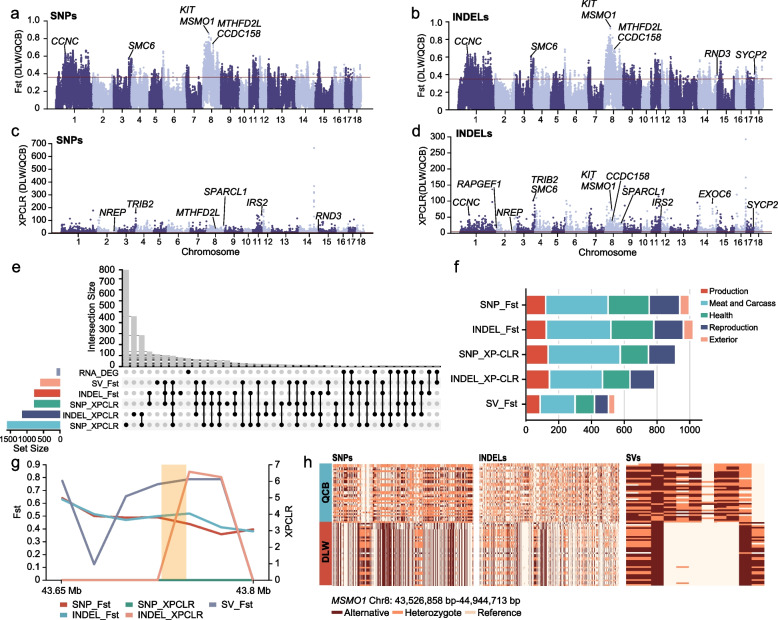
Genome-wide screening of candidate genes for intramuscular fat in pigs. **a**–**d** Genome-wide distribution (sliding window = 50 kb, step size = 25 kb) of selective eliminations determined by fixed index (Fst) (SNPs (**a**) and INDELs (**b**)) and XPCLR analysis (SNPs (**c**) and INDELs (**d**)) between DLW and QCB pigs using SNPs (**a**) and INDELs (**b**). **e** Upset plot showing the intersection of differentially expressed genes in the transcriptome analysis of the longissimus dorsi muscle and annotated genes in the selected regions (top 5%). **f** Bar plot showing QTL annotations in the selected eliminated regions. **g** XPCLR values and Fst values of the *MSMO1* gene and its upstream and downstream genomic regions (total 0.15 Mb). **h** Haplotypes of the *MSMO1* gene and its upstream and downstream 200 kb genomic region

Significant differences in intramuscular fat content between QCB and DLW pigs were found in a previous study, and the samples used in that study were from the same population as those used in this study [18]. We used two statistical methods (Fst and XPCLR) to search for regions in the genome, and regions with values in the top 5% were considered candidate genomic regions (Fig. 4a–d; Fig. S3).

In this study, SNPs-based genome scanning results showed that the Fst value of the variant set after integration was significantly higher than that before integration (The average difference was 0.004458 ± 3.9 × 10⁻^5^, t = 114.836, *P* < 0.0001), and the XPCLR value also significantly improved (mean difference 0.083877 ± 2.4 × 10⁻^2^, t = 3.483, *P* = 0.0005). This trend indicates that after integrating the mutation set, the differentiation signal of SNPs among populations is stronger. In the genome scan of INDELs, we observed that the Fst value of the integrated variant set also increased (The average difference was 0.015884 ± 1.27 × 10⁻^4^, t = 125.036, *P* < 0.0001), while the XPCLR value decreased (The mean difference is −0.217820 ± 1.61 × 10⁻^2^, t = −13.550, *P* < 0.0001). By annotating the top 5% of candidate regions, we counted the number of windows, the number of annotated candidate genes, the percentage of overlapping abnormal genes before and after integration, and QTL annotation entries (Table 2). The results of SNPs-based genome scanning showed that the number of windows and the number of annotated candidate genes corresponding to the integration strategy increased. The INDELs-based Fst results also show a similar trend, indicating that the integration process may help reveal more candidate regions. The results of INDELs-based XPCLR analysis showed that although the number of windows increased after integrating the variant set, the number of candidate genes decreased. In addition, INDELs-based Fst analysis also showed that the percentage of overlapping abnormal genes before and after integration was significantly lower than other gene types. This phenomenon suggests that the sensitivity of the XPCLR method may vary when dealing with INDELs variants or may be affected by specific selection signal patterns.

**Table 2:** Summary of outlier windows, outlier genes, overlapping genes, and QTL annotations

Mutation type	Method	Outlier windows	Outlier genes	Percentage of overlapping genes	Number of QTL annotation entries
SR-SNPs	Fst	4,530	775	96.00%	1,008
Merged-SNPs	Fst	4,530	781	95.26%	998
SR-INDELs	Fst	4,515	711	2.95%	943
Merged-INDELs	Fst	4,530	786	2.67%	1,022
SR-SNPs	XPCLR	4,260	1,438	62.66%	994
Merged-SNPs	XPCLR	4,363	1,605	56.14%	994
SR-INDELs	XPCLR	3,434	1,264	43.59%	874
Merged-INDELs	XPCLR	3,360	1,150	47.91%	846
SVs	Fst	2,272	595	NA	536
SVs	XPCLR	19	0	NA	0

We annotated QTL for the first 5% of the selected scanned areas. The results showed that QTL related to meat and carcass had the highest detection rate (Fig. 4f). To identify genes that specifically regulate intramuscular fat deposition, we analyzed RNA-seq data from the longissimus dorsi muscle of QCB pigs and DLW pigs [18]. By integrating genes identified by selection signals and transcriptome screening for DEGs related to fat metabolism, we identified 14 candidate genes most likely to affect IMF in pigs (Fig. 4e; Table S10).

EigenGWAS was conducted using three types of genetic variations (SNPs, INDELs, and SVs). The results of PCA analysis indicated that the first eigenvector could successfully separate QCB pigs and DLW pigs into distinct groups. However, the second eigenvector did not show a significant effect in distinguishing these two pig breeds and failed to achieve group separation (Fig. S4a, d, g). Therefore, the first eigenvector was used as a phenotype for EigenGWAS, which identified a total of 5,115 significant SNPs, 2,149 significant INDELs, and 62 significant SVs (Fig. S4b, c, e, f, h, i). Unlike the results from Fst and XPCLR analyses, these significant loci were almost exclusively concentrated on chromosome 8. After annotation, it was found that they, along with the selection signals identified previously, jointly pointed to the genes *KIT* and *MSMO1* (Table S11).

We observed that both the Fst values and the XPCLR scores in the *MSMO1* gene region are generally high, yet the XPCLR scores based on SNPs in this region are all 0 (Fig. 4g). We constructed haplotypes of the 200 kb flanking genomic region of the *MSMO1* gene and found a consistent haplotype pattern (Fig. 4h). Subsequently, we screened for variants within the exons of the *MSMO1* gene and found a missense mutation (Chr8:43,738,941; *rs81509043*). This mutation was all Homozygous reference in the Large White pig population, but only 56% was a homozygous reference in the Qinchuan Black pig population (41% were heterozygous variant and 3% were homozygous variant). The differential expression of this gene may be due to differences in protein structure and function caused by exon variations.

### *MSMO1* promotes pig intramuscular adipocyte proliferation and inhibits lipogenic differentiation

**Fig. 5:**
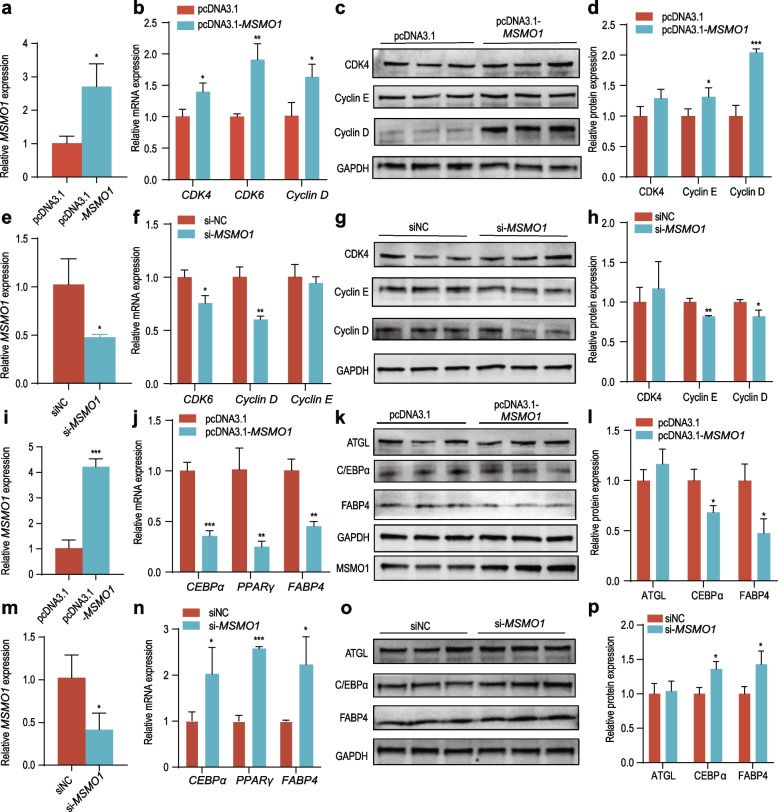
*MSMO1* promotes the proliferation of pig intramuscular adipocytes and inhibits lipogenic differentiation. **a** *MSMO1* overexpression efficiency assay in proliferating cells. **b** mRNA expression of proliferation-related genes after overexpression of *MSMO1*. **c** and **d** Protein levels of proliferation-related genes after overexpression of *MSMO1*. **e** *MSMO1* interference efficiency assay in proliferating cells. **f** Proliferation-related gene mRNA expression after interference with *MSMO1*. **g** and **h** Proliferation-related gene protein levels after interference with *MSMO1*. **i** *MSMO1* overexpression efficiency assay in induced differentiated 8 d cells. **j** mRNA expression of differentiation-related genes after overexpression of *MSMO1*. **k** and **l** Protein levels of differentiation-related genes after overexpression of *MSMO1*. **m** *MSMO1* interference efficiency assay in induced differentiated cells. **n** Differentiation-related gene mRNA expression after interference with *MSMO1*. **o** and **p** Protein levels of differentiation-related genes after interference with MSMO1. *P < 0.05, **P < 0.01, ***P < 0.001

To further investigate the role of *MSMO1* on adipogenic differentiation, we selected pig intramuscular adipocytes for subsequent experiments. RT-qPCR results showed that mRNA expression of proliferation-related genes *CDK4*, *CDK6* and cyclin D was significantly increased by overexpression of *MSMO1* (Fig. 5a and b). Protein expression of proliferation-related genes was also significantly increased in the treated group compared to the control group (Fig. 5c and d). We simultaneously inhibited with the gene using siRNA in the cells, which showed a significant decrease in the expression of some proliferation-related genes (Fig. 5e–h). The above results suggest that *MSMO1* promotes the proliferation of pig intramuscular adipocytes.

Next, we explored the effect of *MSMO1* on adipocyte lipogenic differentiation. The results showed that after overexpressing *MSMO1* and inducing differentiation for 8 d, the mRNA expression of genes related to adipogenic differentiation, *C/EBPα*, *PPARγ*, and *FABP4*, was significantly reduced (Fig. 5i and j). Protein expression of *C/EBPα* and *FABP4* showed the same trend (Fig. 5k and l). Inhibition of *MSMO1* showed opposite results, and we found a significant increase in the expression of lipogenic differentiation genes (Fig. 5m–p). Together, these results suggest that *MSMO1* inhibits lipogenic differentiation of pig intramuscular adipocytes.

## Discussion

The Danish Large White pig (DLW) is a widely used lean pig breed, and the Guanzhong Black pig (GZB) is an excellent meat-fat dual-purpose breed developed in China in the early days. Both breeds are well-represented in the study of fat deposition. Although there have been comparative studies on pig fat deposition and some fat-related genes have been reported, most of these studies are based on mapping short-read data onto reference genomes to analyze differences between species or different physiological stages [44, 45]. However, reference genomes can introduce mapping biases that significantly affect downstream analysis and inference. Therefore, although the traditional single reference genome method occupies an important position in the field of genotyping, its limitations have prompted the development of new genotyping tools and strategies. A recent three-spined stickleback study demonstrated that the use of local reference genomes can provide higher mapping efficiency and reduce the proportion of missing data, thereby increasing the confidence of downstream analyses [16]. In addition, it is generally accepted in the genetics community that the parental genome contains the complete genomic information of the offspring. In a previously reported study of melon populations, a graph-based approach using parental genomes was able to improve variant calling rate and calling accuracy [17]. However, this strategy has not been reported in livestock.

This study provided a more complete and accurate genetic variation panel for offspring samples based on an integration strategy, and ultimately identified candidate genes associated with intramuscular fat (IMF) content in pigs after analysis. Compared with the traditional single-reference genome method, the comprehensiveness and accuracy of variant detection are significantly improved after integrating the genomes of both parents. Specifically, this strategy integrates data from the reference genome and parental genome, resulting in a markedly increased number of detected variants (including SNPs, INDELs, and SVs), with an even greater increase observed after quality control. There may be two reasons for this. One is that the typing panel used by the Pan method has a high similarity with the variants in the population sample; the other is that the PanGenie algorithm itself is more sensitive to variants in different samples, thereby reducing false positives caused by differences between samples. Furthermore, the detection rate of non-reference genotypes has improved, and the characteristics of variants have shown greater consistency, which further substantiates the reliability of the integration strategy.

We have identified an interesting phenomenon: when based on SNPs, the *MSMO1* gene region along with its 150 kb upstream and downstream regions exhibit high Fst values, while the XPCLR score is 0. This phenomenon may be explained by the intense selective pressure this gene underwent during the early stages of breeding, leading to its fixation and subsequent population differentiation (high Fst). However, the XPCLR method becomes ineffective due to the absence of allele frequency gradients, and it fails to detect any recent selection signals. In particular, the integrated selection signal analysis identified more differential windows and differential genes, identifying 14 of the most promising candidate genes: *MSMO1*, *SMC6*, *CCDC158*, *KIT*, *CCNC*, *IRS2*, *TRIB2*, *NREP*, *SPARCL1*, *EXOC6*, *RAPGEF1*, *SYCP2*, *RND3* and *MTHFD2L* (Table S10). The *KIT* gene is a crucial determinant of variations in pig coat color [46, 47]. In Yorkshire pigs, after eliminating the duplication and splicing mutations in the *KIT* gene, although there was no change in coat color, the meat color improved and the red blood cell count increased [48]. This suggests that *KIT* may indirectly influence meat color by affecting erythropoiesis rather than directly participating in melanin synthesis, providing new clues for unraveling the link between coat color genes and meat quality. Chinese indigenous black pigs often exhibit both black coats and high-quality meat, implying a potential correlation between coat color genes and meat quality traits. In the future, by precisely regulating the *KIT* gene and its interaction network, it may be possible to breed improved pig breeds that retain desirable coat colors while enhancing meat quality. The *MSMO1* gene has been reported to negatively regulate adipogenesis in 3T3-L1 cells and is associated with IMF in chickens [49, 50]. This study further elucidates the dual regulatory functions of *MSMO1*: it stimulates the proliferation of pig primary intramuscular adipocytes while concurrently suppressing their adipogenic differentiation. Remarkably, most of these genes were screened in pigs for the first time. This suggests that it is promising to integrate parental genome information for downstream analysis to identify candidate genes.

## Conclusion

In this study, we demonstrated the advantages of integrating biparental genomic information for variant detection and population genomic studies. Compared with traditional single-reference methods, the integration of biparental genomes enhanced the detection of genetic variants, including SNPs, INDELs, and SVs. The integrated strategy improved population structure analysis and revealed clearer clustering patterns. In addition, selection signal analysis identified candidate genes associated with IMF content, including *MSMO1*. Functional validation showed that *MSMO1* promoted intramuscular adipocyte proliferation and inhibited adipogenic differentiation, indicating its potential to be a key regulator of IMF in pigs. Overall, our results highlight the use of strategies integrating biparental genomic information to improve variant detection and enhance our understanding of complex traits, providing new insights for future genetic research in livestock breeding.

## Supplementary Information

Additional file 1: Fig. S1. Variant set obtained by integrating two genotyping strategies (Single-reference genotyping and Graph-based genotyping). Fig. S2. Principal component (PC) analysis (a) and ADMIXTURE analysis (b) based on single reference typing. Fig. S3. Genome-wide distribution (sliding window = 50 kb, step size = 25 kb) of selective eliminations determined by fixed index (Fst) between DLW and QCB pigs using SVs. Fig. S4. EigenGWAS analysis was conducted across the whole genome for 37 QCB pigs and 40 DLW pigs.

Additional file 2: Table S1. Summary information for a total of 108 samples. Table S2. Summary of all primers. Table S3. Statistical summary of the sequences generated by the genome assembly. Table S4. Summary of reference genome and pseudomolecules parental genomes of QCB pig. Table S5. Summary of repetitive sequences in the reference genome and parental genomes of the QCB pig. Table S6. Using Sscrofa 11.1 as the reference genome, this is a summary statistic of INDELs and SVs identified in the parental genomes of Qinchuan Black pigs. Table S7. Summary of the number of variants obtained by the two genotyping methods. Table S8. Summary of genotype frequency statistics. Table S9. Summary of variant types after annotation. Table S10. Summary of candidate genes identified for intramuscular fat. Table S11. Summary of genes annotated by EigenGWAS.

## Data Availability

Data are available from the corresponding author on reasonable request.

## Abbreviations

C/EBPα: CCAAT/enhancer binding protein alpha
CCS: Circular consensus sequencing
CDK4: Cyclin-dependent kinase 4
CDK6: Cyclin-dependent kinase 6
cDNA: Complementary deoxyribonucleic acid
DEGs: Differentially expressed genes
DEXA: Dexamethasone
DLW: Danish Large White pig
DMEM: Dulbecco’s Modified Eagle’s Medium
FABP4: Fatty acid binding protein 4
Fst: Fixation index
GZB: Guanzhong Black pig
IBMX: 3-Isobutyl-1-methylxanthine/Isobutylmethylxanthine
IM: Intramuscular
IMF: Intramuscular fat
INDELs: Insertions and deletions
LINE: Long interspersed nuclear element
MAF: Minor allele frequency
mRNA: Messenger ribonucleic acid
MSMO1: Methylsterol monooxygenase 1
PBS: Phosphate buffer saline
PCA: Principal component analysis
PPARγ: Peroxisome proliferator-activated receptor gamma
PVDF: Polyvinylidene difluoride
QCB: Qinchuan Black pig
RIPA: Radioimmunoprecipitation assay
RNA: Ribonucleic acid
RT-qPCR: Real-time quantitative polymerase chain reaction
siRNA: Small interfering RNA
SNPs: Single nucleotide polymorphisms
SVs: Structural variants
TBST: Tris-buffered saline + Tween
TEs: Transposable elements
XPCLR: Cross-population composite likelihood ratio
