# Supplementary material for: Integrating parental genomes to reduce reference bias and identify intramuscular fat genes in Qinchuan Black pigs
Source: J Anim Sci Biotechnol. 2025 Jul 20;16:104. doi: 10.1186/s40104-025-01236-3 (PMC12276683; doi:10.1186/s40104-025-01236-3)
Supplement: Supplementary file 1 — Additional file 1: Fig. S1. Variant set obtained by integrating two genotyping strategies (Single-reference genotyping and Graph-based genotyping). Fig. S2. Principal component (PC) analysis (a) and ADMIXTURE analysis (b) based on single reference typing. Fig. S3. Genome-wide distribution (sliding window = 50 kb, step size = 25 kb) of selective eliminations determined by fixed index (Fst) between DLW and QCB pigs using SVs. Fig. S4. EigenGWAS analysis was conducted across the whole genome for 37 QCB pigs and 40 DLW pigs. [file 40104_2025_1236_MOESM1_ESM.docx]

**
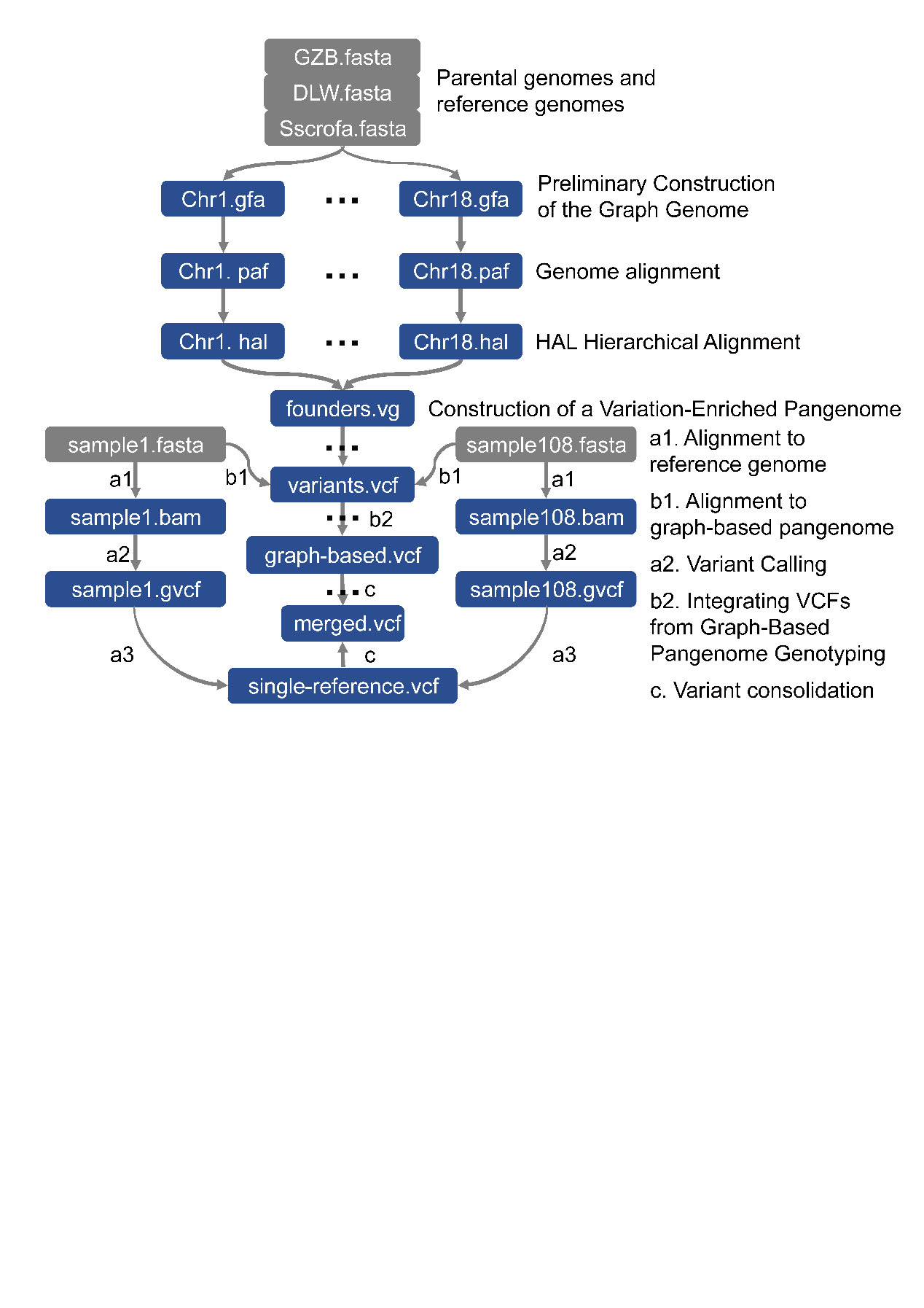
Fig. S1.** Variant set obtained by integrating two genotyping strategies (Single-reference genotyping and Graph-based genotyping). Files are displayed in blocks, with each step labeled on the right. Gray indicates input files.

**
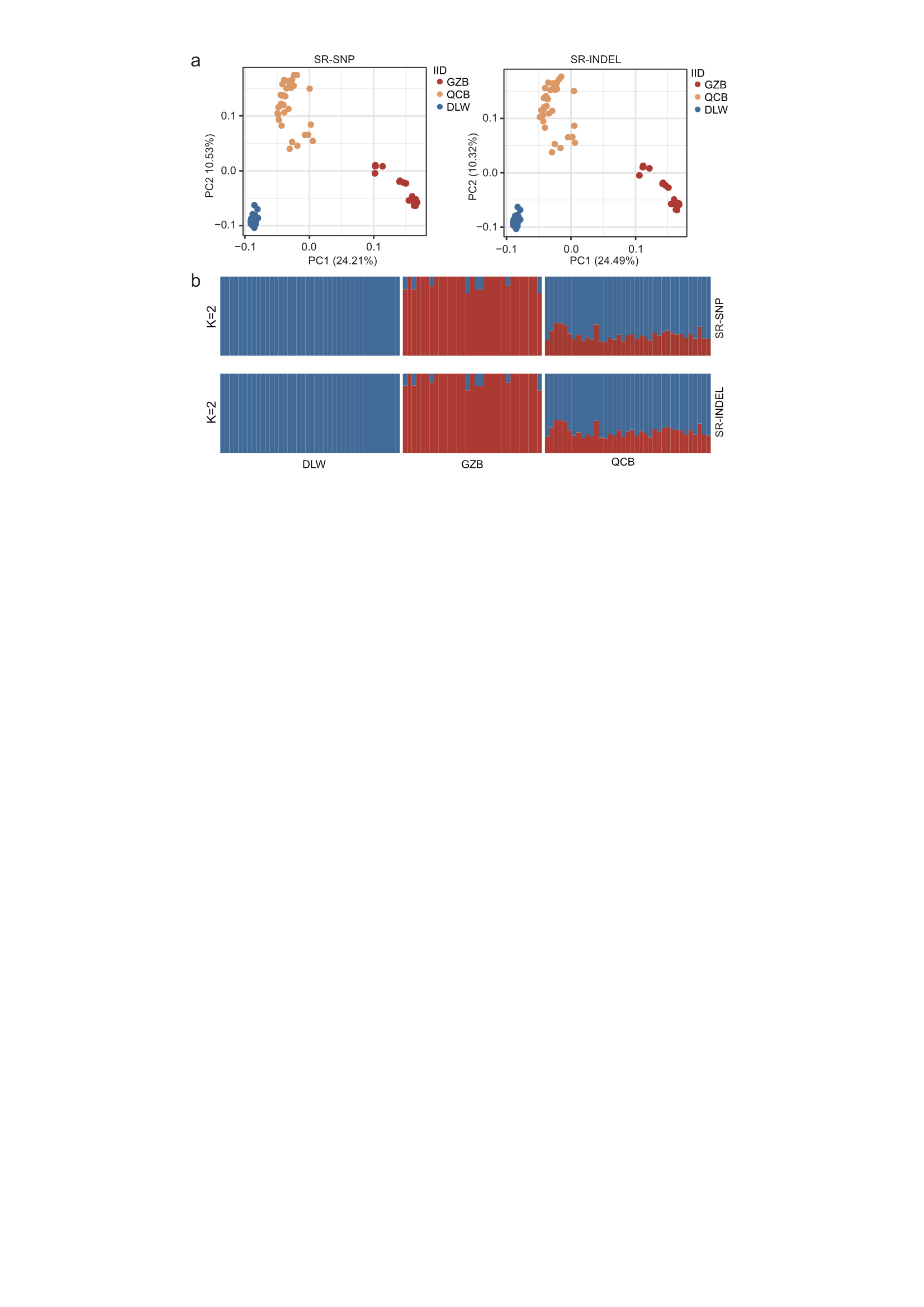
Fig. S2.** Principal component (PC) analysis (**a**) and ADMIXTURE analysis (**b**) based on single reference typing.

**
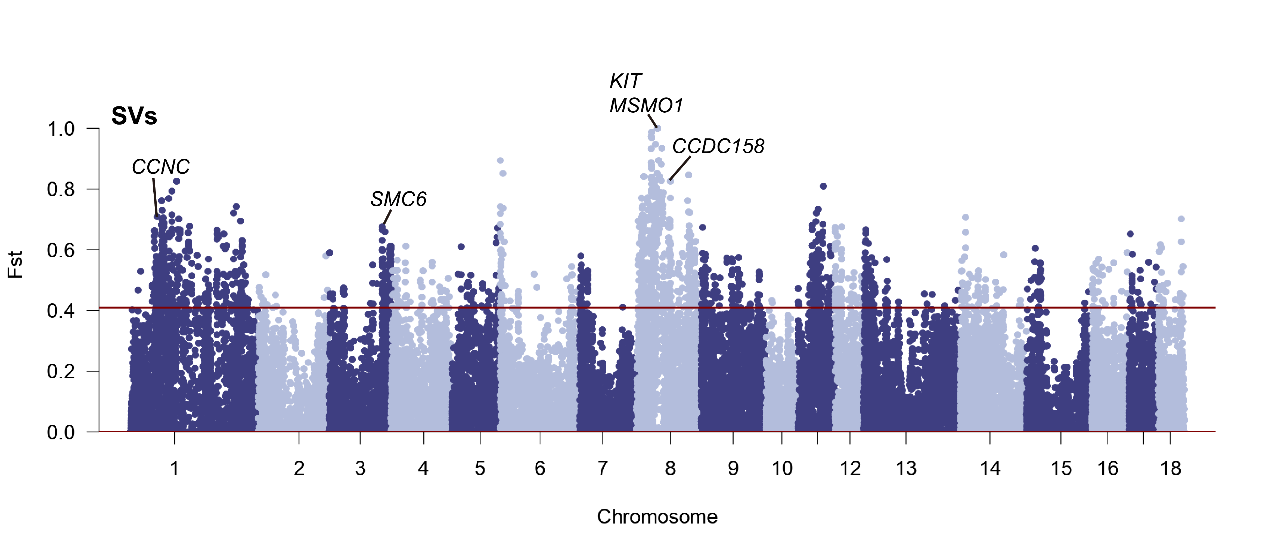
**

**Fig. S3.** Genome-wide distribution (sliding window = 50 kb, step size = 25 kb) of selective eliminations determined by fixed index (Fst) between DLW and QCB pigs using SVs.


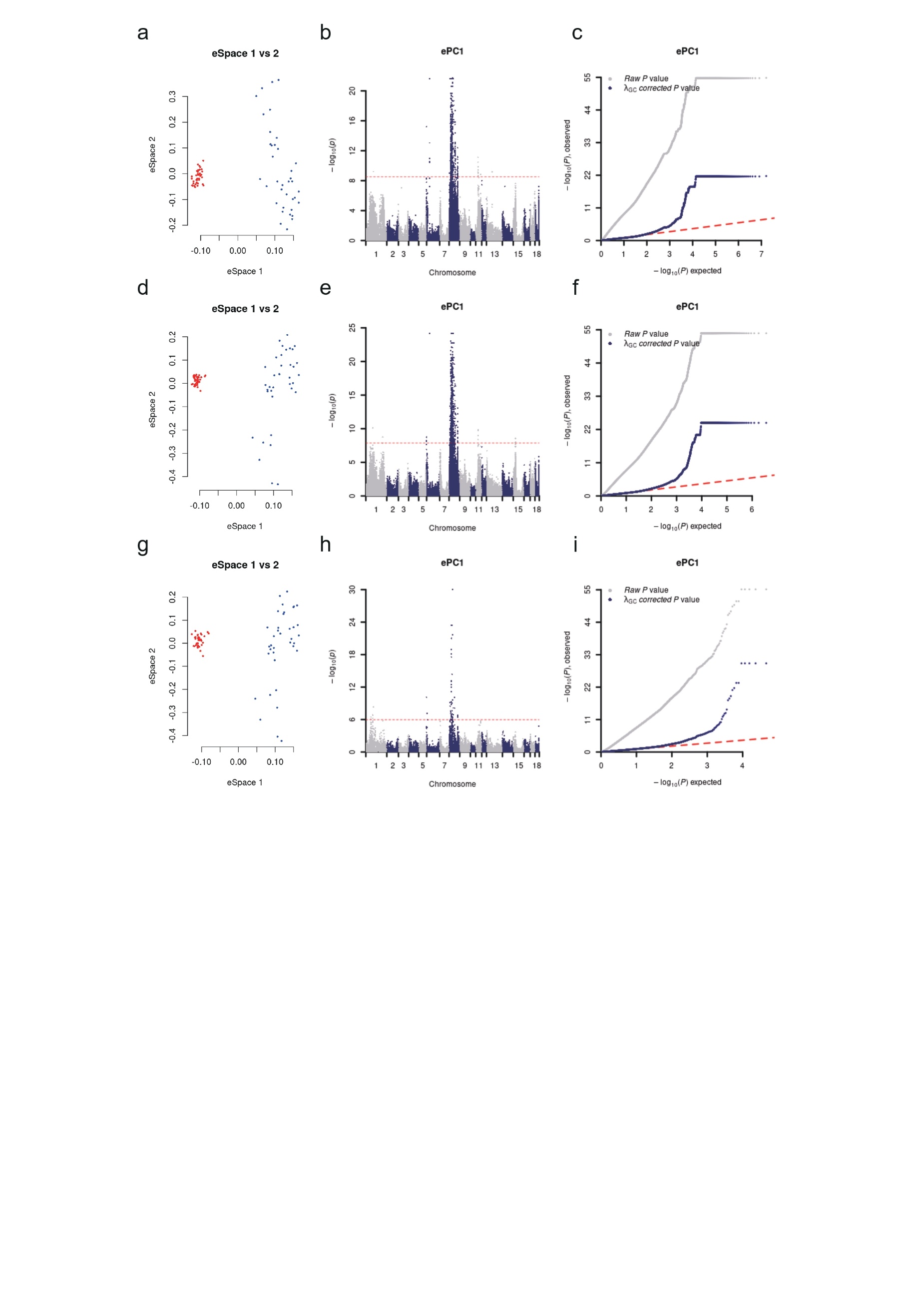


**Fig. S4.** EigenGWAS analysis was conducted across the whole genome for 37 QCB pigs and 40 DLW pigs. The red line represents the 0.05/*n* threshold, with eigenvector 1 used as a covariate. **a-c** The results of EigenGWAS analysis based on SNPs: **a** is a PCA plot, **b** is a Manhattan plot, and **c** is a QQ plot. **d-f** The EigenGWAS analysis results based on INDELs: **d** is a PCA plot, **e** is a Manhattan plot, and **f** is a QQ plot. **g-i** The EigenGWAS analysis results based on SVs: **g** is a PCA plot, **h** is a Manhattan plot, and **i** is a QQ plot.
